# Development and validation of the General attitude towards Medication Questionnaire (GAMQ)

**DOI:** 10.1186/s40359-024-02108-7

**Published:** 2024-11-07

**Authors:** Kaya J. Peerdeman, Judith Tekampe, Henriët van Middendorp, Antoinette I. M. van Laarhoven, Ralph C. A. Rippe, Madelon L. Peters, Andrea W. M. Evers

**Affiliations:** 1 Health, Medical and Neuropsychology Unit, Institute of Psychology, Faculty of Social and Behavioural Sciences, Leiden University, Wassenaarseweg 52, Leiden, 2333 AK The Netherlands; 2 Leiden Institute for Brain and Cognition, Leiden University, Wassenaarseweg 52, Leiden, 2333 AK The Netherlands; 3 Department of Medical Psychology, Radboud University Medical Center, Nijmegen, The Netherlands; 4 Research Methods and Statistics, Institute of Education and Child Studies, Leiden University, Leiden, The Netherlands; 5 Department of Clinical Psychological Science, Maastricht University, Maastricht, The Netherlands; 6 Department of Psychiatry, Leiden University Medical Center, Leiden, The Netherlands

**Keywords:** Medication, Attitude, Questionnaire development, Expectancies, Placebo effects, Nocebo effects

## Abstract

**Background:**

Attitudes towards medication can affect treatment outcomes and adherence through mechanisms such as placebo and nocebo effects. Questionnaires assessing both negative and positive attitudes towards medication in general, which can be used across a variety of settings and in both patient and non-patient samples, are however lacking. To fill this gap, we developed and validated the General Attitude towards Medication Questionnaire (GAMQ).

**Methods:**

Items were selected and adapted from existing questionnaires by a group of experts. Validation of the original Dutch version took place in 4 samples: 2 recruited from the general population (*n* = 508; *n* = 279) and 2 patient samples (patients with rheumatoid arthritis, *n* = 121; patients with atopic dermatitis, *n* = 70). We evaluated the psychometric properties of the GAMQ by determining the factor structure and its stability across samples, internal consistency, and convergent validity.

**Results:**

The GAMQ contains 6 positive and 6 negatively worded items. A factor structure was observed with three subscales, representing ‘Trust in medication’, ‘Concerns about medication’, and ‘Reluctance to use medication’. The fit of the factor structure was satisfactory across samples, considering classic cut-offs, with an adequate or close to adequate fit. The total scale showed good internal consistency, good convergent validity with related scales (i.e., Beliefs about Medicines Questionnaire - General and a single medication attitude scale), and concurrent validity as reflected in associations with expectations about pain- and itch-relieving medication. It was not consistently or strongly associated with demographic or health-related characteristics.

**Conclusions:**

The newly developed GAMQ showed satisfactory psychometric properties in a variety of populations, although limitations should be considered. The GAMQ is the first scale to assess both positive and negative attitudes towards medication in general, providing indicators of Trust, Concerns, and Reluctance regarding medication. The scale may be an informative measure for predicting treatment outcomes and adherence, as well as placebo and nocebo effects in diverse samples.

**Supplementary Information:**

The online version contains supplementary material available at 10.1186/s40359-024-02108-7.

## Background

Attitudes towards and beliefs about medication have been identified as important predictors of treatment outcomes and, partially as a consequence of that, adherence. Medication attitudes and beliefs entail opinions or perceptions one may have about the benefits, harms, and use of medication. Where attitudes may pertain more to opinions on personal use of medication and beliefs to general opinions about medication, these distinctions are not explicitly made in the literature [1–4]. We use the term attitudes towards medication to reflect both. Knowledge of medication attitudes may prove instrumental in addressing limited beneficial treatment outcomes, harmful side effects, and non-adherence, which are known major problems in healthcare worldwide, linked with poor health and high healthcare costs [5–7].

Negative attitudes towards medication have been associated with side effects [7–10]. This association may be explained by nocebo effects (i.e., negative treatment outcomes that cannot be explained by a treatment’s active components; the negative counterpart of placebo effects) [9, 10]. Negative attitudes towards medication, including concerns about the potential for dependence on medication and harmful long-term effects, have also been found to be predictive of non-adherence [7, 11, 12].

Positive attitudes towards medication may increase medication effectiveness [10, 13, 14], akin to how positive outcome expectancies of a specific treatment can contribute to placebo effects [15, 16]. Having a positive attitude towards medication in general, trusting the effectiveness of the medication, and being convinced that the treatment is needed, has also been associated with reduced side effects [7] and increased adherence [12, 17]. Ultimately, fostering positive attitudes towards medications could help in optimizing the effects of pharmacotherapy and preventing nocebo-induced side effects and non-adherence. Notably, positive and negative attitudes are not necessarily two ends of the same spectrum. As with positive and negative affect, one can hold both positive and negative attitudes simultaneously and they can have both unique and interactive effects [18, 19].

Despite these indications that both negative and positive attitudes towards medication are of importance, current research focuses more on the impact of negative attitudes towards medication [7–10]. This focus on negative attitudes is also reflected in the instrument currently available to assess *general* attitudes towards medication, the Beliefs about Medicines Questionnaire (BMQ-G [2]). Despite its clear value, the assessment of only negative beliefs along with the phrasing of items as general statements (e.g., “Doctors prescribe too many medicines”), might steer respondents towards a negative attitude. Another scale, assessing the related concept of sensitivity to medicines [Perceived Sensitivity to Medicines scale, 20], also has a negative focus. Other questionnaires exist that do assess positive as well as negative attitudes, but these are concerned with how patients view their own medications, i.e., with attitudes towards the *specific* medication a patient is currently using such as analgesic or anti-psychotic medication [Pain Medication Attitude Questionnaire, 1, BMQ-Specific Concerns and Necessity scales, 2, 3, Drug Attitude Inventory-10, 4, 21]. Moreover they may not assess positive attitudes comprehensively. These scales are as such highly valuable in specific contexts but do not allow comparisons across diverse settings and samples.

A novel questionnaire is needed to enable research into both negative and positive attitudes towards medication in general across a variety of settings and samples, including non-patient samples. The use of such a scale in both experimental and clinical research can provide a more balanced and comprehensive understanding of how attitudes towards medication predict treatment outcomes and adherence than is possible to date. The current paper describes the development of such a questionnaire, the General Attitude towards Medication Questionnaire (GAMQ), and the evaluation of its psychometric properties in both healthy and patient samples (rheumatoid arthritis, RA, and atopic dermatitis, AD). Patients with RA and AD often experience chronic pain or itch for which many use medication for an extended period of time. Physical symptoms, particularly pain, but also itch, are known for their sensitivity to placebo and nocebo effects [16, 22–24]. Since these effects have previously been found to be predicted by attitudes towards medication [9, 10], it seems relevant to study these patient groups. We evaluated the psychometric properties of this newly developed GAMQ by determining (1) the factor structure and (2) the internal consistency in two general and two patient samples. Also, to provide first indications of the GAMQ’s usefulness in research, (3) its convergent validity (i.e., association with existing medication attitude questionnaire BMQ-G and a single medication attitude scale) and (4) concurrent validity (i.e., association with expected effectiveness and side effects of medication) were investigated. We hypothesized that the GAMQ would correlate negatively with the BMQ-G subscales and expected side effects of medication, and positively with a single medication attitude visual analogue scale and overall expected effectiveness of medication for pain and itch relief. Finally, to aid the interpretation of the questionnaire scores in future research and add to the information of its potential usefulness in different populations, we explored (5) associations with demographic and health-related characteristics in each of the samples.

## Methods

### Respondents

For the validation of the GAMQ, data collected as part of four larger studies were used. The studies differed with regard to the overarching aims and availability of additional data. Data from a general sample (Gs1) [25] and samples of patients with rheumatoid arthritis (RAs) and atopic dermatitis (ADs) were obtained from studies investigating expectations about different routes of medication administration (in the RAs sleep quality and circadian rhythm were also investigated). Data from a second general sample (Gs2) were obtained from a study investigating the induction of pain medication expectations by verbal suggestion and investigating mental imagery abilities through questionnaires [26]. Although no formal power analyses were conducted a priori, the literature suggests that the samples sizes should generally be sufficient for the analyses of the factor structure and correlations [27, 28]. The Psychology Research Ethics Committee of Leiden University approved the studies in the general samples (Gs1: PREC15-0828/33, Gs2: CEP16-0226/99). The Medical Ethics Committee of the Leiden University Medical Center approved the studies in the patient samples (RAs: P15.233, ADs: P15.332). All respondents were required to provide written informed consent, either digitally or on paper. All collected data that is of relevance for the evaluation of the GAMQ is described in the current manuscript. A brief overview of available data per sample is presented in Table S1 in the Supporting Information. In all cases, the GAMQ was administered towards the end of the survey, after the assessments relevant to the other study aims mentioned above.

#### General sample 1 (Gs1)

A general sample that was representative of the adult Dutch population in terms of gender, age, and province of residence was recruited via an online service (Qualtrics, Provo, UT, USA) in September 2015. Inclusion criteria were being ≥ 18 years and fluency in Dutch. All respondents were asked to fill in a battery of online questionnaires, including the GAMQ, taking approximately 20 min.

#### General sample 2 (Gs2)

A second general sample that was representative of the Dutch population in terms of gender and age was recruited via posters and flyers at various public locations (e.g., library, gym, pharmacy), social media, participant recruitment websites, and personal networks in the Netherlands. Inclusion criteria were being 18 to 65 years and fluency in Dutch. Data collection took place from March to June 2016. Respondents filled in a battery of online questionnaires, including the GAMQ, taking approximately 35 min via Qualtrics.

#### Rheumatoid arthritis sample (RAs)

Patients who fulfilled the 1987 ACR criteria for rheumatoid arthritis were recruited from the Leiden Early Arthritis Clinic cohort [29], a population-based prospective cohort. The ACR criteria have high sensitivity and specificity to differentiate between RA and non-RA rheumatic diseases [30]. Further inclusion criteria were being ≥ 18 years and fluency in Dutch. Patients were approached via email and could choose whether they wanted to fill out the battery of questionnaires via Qualtrics or using paper and pencil. Data collection took place in October 2015. Filling out the questionnaires took approximately 45 min.

#### Atopic dermatitis sample (ADs)

Patients with AD who visited the Department of Dermatology of the Leiden University Medical Center in the preceding year were approached via regular post from January to March 2016. Next to a diagnosis of atopic eczema, inclusion criteria were being ≥ 18 years and fluency in Dutch. They could choose whether they wanted to fill out the battery of questionnaires via Qualtrics or using paper and pencil. Completing the questionnaires took participants around 30 min.

### Development of the General attitude towards Medication Questionnaire (GAMQ)

To assess both positive and negative attitudes toward medication in general, we developed the General Attitudes towards Medication Questionnaire (GAMQ). Our goal was to develop a brief and easy to understand questionnaire that can be used across diverse patient and non-patient samples. To ensure a balanced measure of attitudes, positively and negatively worded items had to be equal in number and were to be presented alternately. To enable the use of the questionnaire in different populations including healthy respondents and patients, items had to be about medication in general rather than specific medication. To prevent biasing respondents toward a certain direction, it was further predetermined that the items should clearly reflect a personal view (e.g., “I think…”) rather than a general statement (e.g., “Doctors prescribe too many medicines”).

Items for the GAMQ were adapted from other available questionnaires measuring attitudes towards and beliefs about medication or were newly developed. Pre-selection of suitable items was done by KP and JT from the general and specific scales of Beliefs about Medicines Questionnaire [BMQ-G, BMQ-S, 2], the Pain Medication Attitude Questionnaire [PMAQ; 1], the Drug Attitude Inventory [DAI, 4, 21], and a study-specific attitude towards pain medication scale [3]. Items were to cover attitudes regarding all aspects considered relevant (i.e., effectiveness, trust, necessity, safety, use, side effects). If these were not found in the existing questionnaires, new items were formulated. The wording of the items was adapted to fulfill the predetermined criteria (i.e., reflecting attitudes towards medication in general, balance in positive and negative wordings, brief, easy to understand, and reflecting a personal view). The pre-selected, adapted, and novel items were discussed within the team of experts in Medical and Health Psychology (AE, AvL, MP, FK, JT, and KP) on multiple occasions. This process resulted in the final selection of 12 items for validation (see Table 1). Six items reflect a positive view of medication (e.g., “I think that medication can help with my symptoms”) and 6 reflect a negative view of medication (e.g., “I am concerned about the side effects of medication”). The instructions for participants on how to fill in the GAMQ were adapted from the BMQ-G [2]. As in the BMG-G, each item is rated on a 5-point Likert scale, ranging from 1 (‘strongly disagree’) to 5 (‘strongly agree’) [2]. To obtain the total score for the GAMQ, negatively worded items are reverse scored before summing all items, so that higher scores reflect a more positive attitude towards medication. Scoring of the subscales determined during the factor analyses is described in the Results section.

**Table 1:** The English translation of the General attitude towards Medication Questionnaire (GAMQ) including instruction and response options

We want to ask you some questions about what you think about medication in general. We are interested in your views about medication that is available on prescription by a doctor.* Please indicate to what extent you agree or disagree with the following statements. There are no right or wrong answers. We are interested in your views.					
	Strongly disagree	Disagree	Uncertain	Agree	Strongly agree
1) I am concerned about the side effects of medication.	o	o	o	o	o
2) I think that medication can help with my symptoms.	o	o	o	o	o
3) I am afraid that medication has a harmful effect on my body.	o	o	o	o	o
4) If I have symptoms, I readily take medication for them.	o	o	o	o	o
5) I find it unnatural to take medication.	o	o	o	o	o
6) I trust that it is safe to take medication.	o	o	o	o	o
7) I only take medication if there is absolutely no other option.	o	o	o	o	o
8) I have no problem with taking medication if I have symptoms.	o	o	o	o	o
9) I would rather endure my symptoms than take medication.	o	o	o	o	o
10) I trust in the effectiveness of medication.	o	o	o	o	o
11) I am afraid of becoming addicted if I take medication for an extended period.	o	o	o	o	o
12) For me the advantages of medication outweigh the disadvantages.	o	o	o	o	o

*Note* The GAMQ was formulated and tested in Dutch (see Table S2). The English version was obtained by forward-backward translation. * This sentence is slightly modified from the original Dutch version, as we realized post hoc that the original sentence (which translates as “We are interested in your views about medication that you use on prescription by your doctor”) could be misinterpreted as indicating that we are interested in their attitudes towards medications that have been specifically prescribed to them by their own doctor, while were are interested in all medications that are available upon prescription by any doctor. For future research, we recommend to use this adjusted version

The GAMQ was formulated and tested in Dutch (see Table S2). The English version was obtained by forward-backward translation. The initial forward translation from Dutch to English was done by a native speaker and certified translator in collaboration with the authors JT and KP. After consensus was achieved about the first English version, native Dutch author HvM, who was blinded for the original Dutch version, translated the first English version back to Dutch. Comparison of this backward translation to the original Dutch questionnaire and the first English translation by the translator and authors JT and KP led to minor adjustments in the final English translation provided in Table 1.

### Materials and measures

#### General beliefs about medication

Beliefs about medication in general were measured using the Beliefs about Medicines Questionnaire – General [BMQ-G, 2] in all of the investigated samples. The BMQ-G consists of two subscales, the General-Harm and the General-Overuse scales, measuring general beliefs about the harmfulness of medication (e.g., ‘All medicines are poisons’) and doctor’s over-prescription of medication (e.g., ‘Doctors use too many medicines’), respectively. Each of the scales consists of 4 items that are rated on a 5-point Likert scale, ranging from 1 (‘strongly disagree’) to 5 (‘strongly agree’). Summing these items per scale results in scores ranging from 4 to 20, with higher scores reflecting more negative beliefs. The BMQ-G has been found to be a useful tool across medical conditions and cultures, correlating with medication adherence [31]. Internal consistency as indicated by Omega for the General-Harm subscale ranged from ω_t_ = 0.68 (questionable) in RAs to ω_t_ = 0.74 (acceptable) in Gs1 and ADs. For the General-Overuse subscale, Omega was acceptable in all samples, ranging from ω_t_ = 0.74 in ADs to ω_t_ = 0.79 in Gs1.

#### Medication attitude visual analogue scale (VAS)

Respondents of all samples were also asked to rate their general attitude towards medication (‘How do you think about medication in general?’) on a visual analogue scale (VAS) ranging from 0 (‘very negatively’) to 100 (‘very positively’) that was newly created for this research.

#### Expectations regarding medication

Participants of the Gs1, RAs, and ADs rated their expectations about the effectiveness for pain and itch relief as well as about side effects of different routes of medication administration (oral, injection, and topical routes) on visual analogue scales ranging from 0 (‘not at all’) to 100 (‘very much’) [25]. For the current study, VAS scores of the different routes of medication administration were averaged, resulting in three scores reflecting (1) the expected effectiveness of medication for pain relief, (2) the expected effectiveness of medication for itch relief, and (3) the expected side effects of medication.

#### Demographic characteristics

All respondents were asked to provide information about their age, gender, marital status, education level, religious affiliation, nationality, and fluency in Dutch language.

#### Health-related characteristics

Respondents were asked whether they were currently undergoing treatment for any illness(es). Additionally, they were asked to rate the intensity of currently experienced pain or itch on a VAS ranging from 0 (‘none at all’) to 100 (‘very much’). In the GS2, respondents only rated current pain intensity, on an 11-point numerical rating scale with identical anchors as the VAS.

Health-related quality of life was measured using the Short Form-12 [SF-12, 32] in the Gs1 and the Short Form-36 (SF-36) in the RAs and ADs. As the SF-36 contains all items included in the SF-12, responses on the SF-36 were used to compute the SF-12 scores. The SF-12 results in two scores, namely the physical health composite score (PCS), and the mental health composite score (MCS). The PCS consists of physical functioning, role limitations due to physical problems, bodily pain, and general health, and comprises items like ‘During the past 4 weeks, how much did pain interfere with your normal work (including work outside the home and housework)?’. The MCS consists of mental health, role limitations due to emotional problems, social functioning, and vitality, and comprises items like ‘How much of the time during the past 4 weeks have you felt calm and peaceful?’. Scores are calculated based on item response theory, with regression weights standardized for the Dutch population [32]. The SF-12 is widely used across cultures, Dutch population norms are available, and discriminative validity is shown to be good with the scales distinguishing general and patient populations [32].

To assess disease activity and impact in the patient samples, the Disease Activity Score 44 [DAS44, 33] was used in the RAs, while the respondents in the ADs completed the Patient Oriented Eczema Measure [POEM, 34]. The DAS44 is a measure of disease activity in rheumatoid arthritis combining symptoms of tenderness and swelling in 44 joints with the Erythrocyte Sedimentation Rate in mm/hour and a general health assessment expressed on a visual analogue scale ranging from 0 to 100. The DAS44 scores of respondents in the RAs were determined during their last visit at the LUMC (approximately 24 weeks prior to filling in the questionnaire) which were made available for use in this study from the Leiden Early Arthritis Clinic Cohort [29]. High scores on the DAS44 reflect more disease activity, with scores below 1.6 indicating clinical remission. Cut off points for disease activity are low (≤ 2.4), moderate (> 2.4 to 3.7), and high (> 3.7) [35]. The DAS44 has been extensively validated, demonstrating scores to be predictive of rheumatoid arthritis disease progression [33]. The POEM was used to measure eczema severity in the ADs. It comprises seven items assessing on how many days of the past week the patient experienced 7 different symptoms of eczema. Each item is rated from 0 (‘no days’) to 4 (‘every day’), resulting in a minimum score of 0 and a maximum score of 28, with higher scores indicating more severe eczema. Additionally, scores are banded into categories as follows: 0 to 2 clear or almost clear from eczema, 3 to 7 mild eczema, 8 to 16 moderate eczema, 17 to 24 severe eczema, and 25 to 28 very severe eczema. The POEM is recommended as the core instrument for measuring atopic dermatitis in clinical trials based on validity, reliability, and feasibility criteria [36]. Internal consistency of the POEM in the current study was good (ω_t_ = 0.89).

#### Quality of the responses

In line with other work from our group [25, 26], several measures were taken to ensure the quality of the responses. For all samples, participants were asked to judge their fluency in Dutch and the questionnaire sets concluded with two items inquiring whether respondents were able to understand the questions asked in the questionnaire and whether respondents had read all questions well before answering. Data of participants who indicated not being able to speak Dutch fluently and of those who indicated not having understood and/or read all or most of the questions were excluded from analyses. The questionnaire sets for both general samples included two additional control questions on which respondents were requested to give a pre-defined answer, to check whether they read the questions well. Data of participants who did not give the requested answer were excluded from analyses. Furthermore, data of participants in both general samples who filled out the total (online) survey in less than 1/3 of the total median time were excluded, as this raised doubts about the reliability of the data. Last, in Gs1 we also excluded data of participants who were over quota in terms of gender, age, or province of residence to optimize representativeness of the general adult Dutch population [25].

### Statistical analyses

Descriptive statistics, univariate analyses of variance (ANOVAs), chi-square tests, and Pearson correlations were calculated using SPSS version 25 (IBM Corporation, Armonk, NY, USA). Factor analyses were run using the Latent Variable Analysis software package (lavaan 0.6-3) in R (version 3.4.3). The significance level for all tests was set at α = 0.05 (two-tailed). For correlations, values around *r* = .10, 0.30, and 0.50 were inferred to indicate small, medium, and large correlations, respectively [37]. For ANOVAs, partial eta squared (η_p_^2^) was calculated as an indicator of the effect size. Cut off values of η_p_^2^ = 0.01, 0.06, and 0.14 were used to indicate small, medium, and large effects, respectively [37]. All correlations and ANOVAs, including post-hoc tests, were tested for significance using simple bootstrap with 1000 samples and the bias corrected accelerator.

Between-sample differences in medication attitude scores, as well as demographic and health-related characteristics were analyzed using univariate ANOVAs for continuous variables and chi-square tests for categorical variables.

The factor structure of the newly developed GAMQ was investigated in all samples using the following procedure [38, 39]. First, an exploratory principal component analysis using varimax rotation was run (while also checking component correlations via oblique rotation) in a random subsample consisting of 60% of Gs1 (training sample) to inform on the general component structure of the GAMQ. Next, the resulting structure was tested by a confirmatory factor analysis in the same training sample with bootstrap (1000 draws), and in the remaining 40% of Gs1 (validation sample), using direct robust estimation to obtain robust standard error estimates. All estimations and statistics were obtained with all variables formally defined as ordinal, using the diagonally weighted least squares on full weight covariance matrix (WLSMV) estimation, the expected information matrix, and the Yuan-Bentler scaled test statistic. In the factor models, the factors were allowed to be correlated and no explicit cross loadings were used in the factor definitions, as we evaluated unique factors/subscales. The generalizability of this factor structure was investigated by confirmatory factor analysis, using 1000 bootstrap draws, in Gs2, and the RAs and ADs. As measures of fit, we calculated the Comparative Fit Index (CFI), the Root Mean Square Error of Approximation (RMSEA; with 90% confidence interval), and the Standardized Root Means Square Residual (SRMR) for each sample. According to the classic cut-offs [40], CFI values close to 0.95 or above, RMSEA values close to 0.06 or below, and SRMR values close to 0.08 or below are considered to indicate adequate fit. However, note that RMSEA is generally not recommended for samples ≤ 250, and that more recent insights also indicate that universal cutoff values would not be appropriate for the specific ordinal models used here. Thus the classic threshold values may not apply to the current conditions, rather the fit indices may offer diagnostic tools for model improvement [41, 42].

As an indicator for internal consistency of the GAMQ, McDonald’s omega total (ω_t_) was computed for the questionnaire as a whole and for all subscales identified by the factor analysis. Benchmark values for ω_t_ were ≤ 0.50: unacceptable, 0.50-0.60: poor, 0.60-0.70: questionable, 0.70-0.80: acceptable, 0.80-0.90: good, and ≥ 0.90: excellent [43]. Also, ω_t_ if each individual item is omitted was reported for the GAMQ total and subscale scores. Furthermore, the distribution of scores and use of each item of the GAMQ was described. As indicators of convergent validity, correlations of the GAMQ total and subscale scores with the BMQ-G General-Harm and General-Overuse subscales and with respondent’s medication attitude VAS score were calculated. To explore concurrent validity of the GAMQ, correlations with expected medication effects were calculated. Finally, correlations of the GAMQ total and subscales with demographic and health variables were calculated. The data and analyses scripts that support the findings of this study are deposited in the online archiving system DataverseNL at 10.34894/HGTM1Y, along with study materials. Due to privacy issues, the data are not shared publicly, but will be made available to individuals upon reasonable request.

## Results

### Sample characteristics and comparisons

**Fig. 1:**
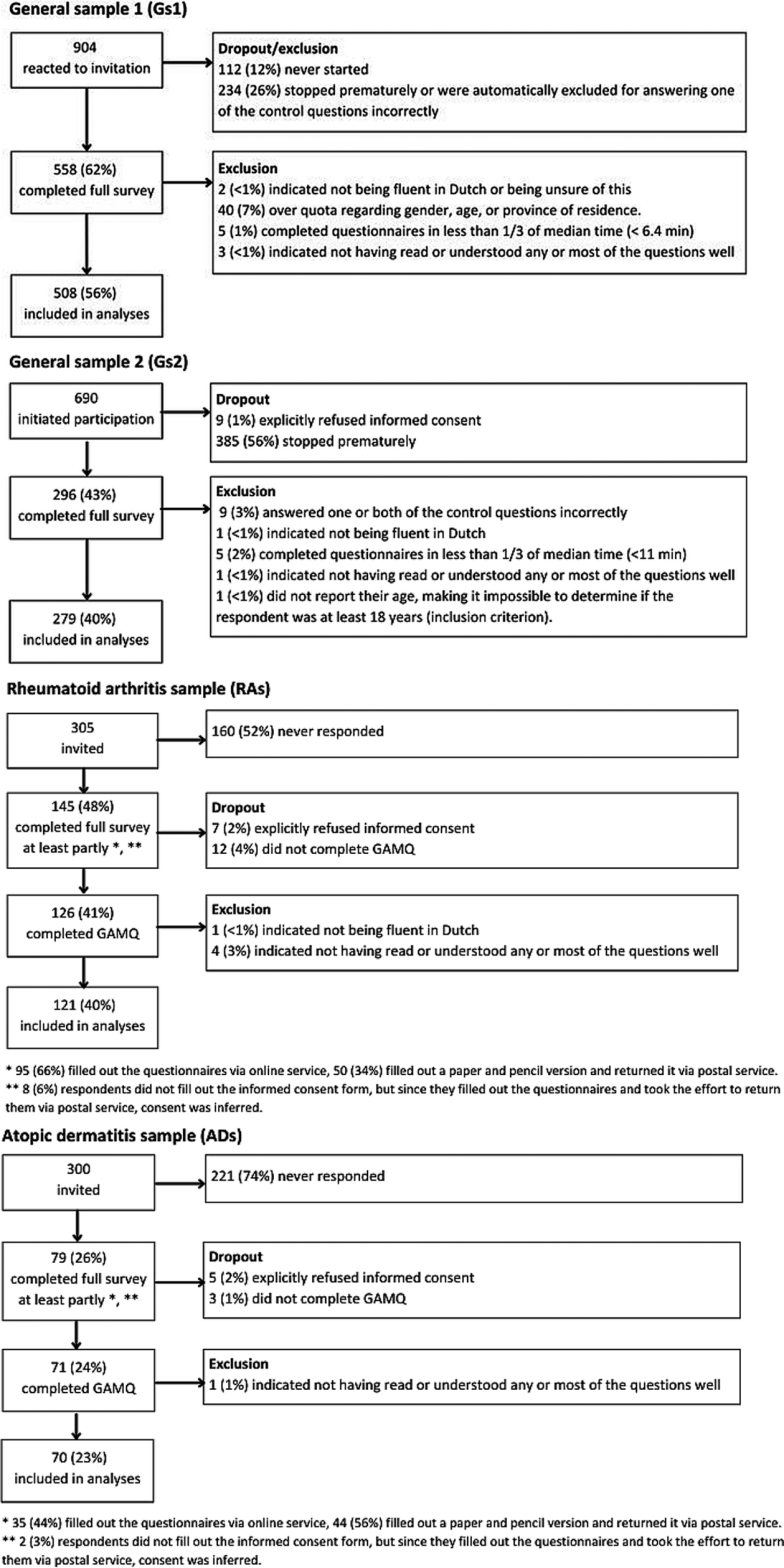
Flow diagrams describing the inclusion of respondents per sample

The inclusion of respondents per sample is described in flow diagrams in Fig. 1. Table 2 provides an overview of the demographic and health-related characteristics per sample, including test statistics of the comparisons between the samples. Regarding demographic characteristics, notable differences, supported by pairwise comparisons, were: (1) the relatively older age of respondents in the RAs; (2) the larger proportion of women in the RAs; (3) the relatively high education level in especially the Gs2, but also the ADs; (4) the relatively high number of respondents who had no religious or ideological affiliation in the Gs2, particularly in comparison to the patient samples; and (5) the somewhat higher proportion of respondents in a relationship in the RAs.

**Table 2:** Demographic and health-related characteristics for each sample

	Gs1 *(n = 508)*	Gs2 *(n = 279)*	RAs *(n = 121)*	ADs *(n = 70)*	Sample comparison *
**Demographic characteristics**					
Age (years) mean (SD), range	47.00 (16.06) 18–75	41.12 (14.18) 18–65	58.63 (13.15) 22–85	47.49 (20.45) 19–82	F(3, 971) = 35.10, *p* < .001, η_p_^2^ = **0.10**
Gender, n *(%) of men*	247 (48.6%)	130 (46.6%)	31 (25.6%)	31 (44.3%)	χ^2^(3) = 21.36, *p* < .001
Educational level, n (%)					χ^2^(3) = 64.28; *p* < .001
Secondary	310 (61.0%)	95 (34.1%)	81 (66.9%)	32 (45.7%)	
Tertiary	198 (39.0%)	184 (65.9%)	40 (33.1%)	38 (54.3%)	
Nationality, n (%)					χ^2^(6) = 7.46; *p* = .281
Dutch	497 (97.8%)	263 (94.3%)	116 (96.7%)	67 (95.7%)	
Other	6 (1.2%)	10 (3.6%)	3 (2.5%)	2 (2.9%)	
Multiple	5 (1.0%)	6 (2.2%)	1 (0.8%)	1 (1.4%)	
Religious or ideological affiliation, n (%)					χ^2^(6) = 39.99; *p* < .001
None	300 (59.1%)	204 (73.1%)	59 (49.2%)	29 (42.0%)	
Christian	178 (35.0%)	65 (23.3%)	57 (47.5%)	37 (53.6%)	
Other	30 (5.9%)	10 (3.6%)	4 (3.3%)	3 (4.3%)	
Marital status, n (%)					χ^2^(3) = 11.40; *p* = .010
Single	175 (34.4%)	89 (31.9%)	22 (18.6%)	20 (28.6%)	
In relationship	333 (65.6%)	190 (68.1%)	96 (81.4%)	50 (71.4%)	
**Health-related characteristics**					
In treatment for long-lasting medical or psychological complaints or diseases (other than RA for RAs and AD for ADs), n (%)	218 (42.9%)	88 (31.5%)	54 (45.5%)	31 (44.3%)	χ^2^(3) = 12.08; *p* = .007
SF-12 PCS, mean (SD)	47.94 (11.46)	*n.a.*	42.91 (10.41)	48.82(9.05)	F(2,696) = 10.99, *p* < .001, η_p_^2^ = 0.03
SF-12 MCS, mean (SD)	46.34 (12.11)	*n.a.*	44.88(11.84)	47.01(9.48)	F(2,696) = 0.95, *p* = .386, η_p_^2^ < 0.01
DAS44, mean (SD)	*n.a.*	*n.a.*	1.67 (0.82)	*n.a.*	*n.a.*
POEM, mean (SD)	*n.a.*	*n.a.*	*n.a.*	13.21 (8.28)	*n.a.*
Current pain intensity, mean (SD)	25.93 (30.40)	18.92 (25.07)	34.85 (30.28)	19.58 (23.77)	F(3,973) = 9.97, *p* < .001, η_p_^2^ = 0.03
Current itch intensity, mean (SD)	12.01 (21.66)	*n.a.*	9.72 (19.28)	52.92 (29.47)	F(2,691) = 105.95, *p* < .001, η_p_^2^ = **0.24**
**Expectancies regarding medication**					
Expected effectiveness of medication for pain relief, mean (SD)	67.85 (13.42)	*n.a.*	62.81 (12.02)	62.50 (14.86)	F(2,671) = 9.35, *p* < .001, η_p_^2^ = 0.03
Expected effectiveness of medication for itch relief, mean (SD)	63.04 (15.37)	*n.a.*	60.66 (14.81)	57.21 (17.93)	F(2, 669) = 4.21, *p* = .014, η_p_^2^ = 0.01
Expected side effects of medication, mean (SD)	48.20 (12.62)	*n.a.*	51.93 (12.36)	48.03 (11.49)	F(2, 665) = 2.97, *p* = .052, η_p_^2^ = 0.01

*Note* Gs1: General sample 1, Gs2: General sample 2, RAs: Rheumatoid Arthritis sample, ADs: Atopic dermatitis sample. Available data are reported, missing values were disregarded from analyses. SF-12 PCS: short form 12 physical component score, SF-12 MCS: short form 12 mental component score, DAS44: disease activity score, POEM: patient oriented eczema measure, n.a.: not assessed, SD: standard deviation

Concerning health-related characteristics, notable differences between the samples were: (1) a relatively small proportion of the respondents in Gs2 was in treatment for long-lasting physical or mental complaints; (2) physical health-related quality of life was somewhat lower in the RAs than in the ADs and Gs1, while mental health-related quality of life did not significantly differ between the samples; (3) current pain was higher in the RAs than all other samples, as can be expected given that pain is a primary symptom of rheumatoid arthritis; (4) likewise, current itch was substantially higher in the ADs than in the Gs1 and the RAs, reflecting that itch is a primary symptom of atopic dermatitis. Furthermore, mean disease activity (DAS44) in the RAs was close to clinical remission. In the ADs, POEM scores indicated the mean eczema severity to be moderate. In addition, regarding medication use, most patients with RA reported they were currently using conventional synthetic disease modifying anti-rheumatic drugs (predominantly methotrexate), many (also) used nonsteroidal anti-inflammatory drugs, and few used biological disease modifying anti-rheumatic drugs for their RA. Most patients with ADs used topical corticosteroids (e.g., hydrocortisone or betamethasone) along with moisturizers, while some (also) used oral antihistamines for their AD. The reported time since start of treatment for their condition ranged from early childhood to recent years in the ADs and from around 25 years ago to recent years in the RAs. Last, there were no substantial differences between expected effectiveness and expected side effects of medication between the samples.

Differences in age, sex distribution, relationship status, and physical health-related quality of life in the RAs may relate to the onset of RA often being later in life and the higher prevalence in women than men [44]. Differences between Gs2 and the other samples in education level, religious or ideological affiliation, and health status may relate to recruitment in Gs2 being partially done via social media and personal networks of the researchers and involved university students.

### GAMQ factor structure

An exploratory principal component analysis on 60% of Gs1 suggested that the GAMQ consists of 3 underlying components; one component comprises items 2, 6, 10, and 12 which represent Trust in medication (i.e., Trust; Eigenvalue = 2.85), the second consists of items 1, 3, and 11 which represent Concerns about medication (i.e., Concerns; Eigenvalue = 2.44), and the third contains items 4, 5, 7, 8, and 9 which represent Reluctance to use medication (i.e., Reluctance; Eigenvalue = 2.13). All items were uniquely allocated (only) to the scale on which they had the highest absolute component loading. Taken together, these three components explained 62% of the total variance in all items. Stricter evaluation using confirmatory factor analysis confirmed the suggested 3-component structure in the 60% training sample as well as in the 40% validation set. The estimated standardized factor loadings obtained by confirmatory factor analysis with bootstrap in the same 60% of Gs1, the remaining 40% of Gs1, Gs1 as a whole, Gs2, RAs, and ADs are presented for each item, grouped by subscale, in Table 3. Residual item (standardized) covariances in the fitted factor model across all samples are nonsignificant (<|1.96|), indicating local independence.

**Table 3:** Items of the GAMQ with standardized factor loadings derived from confirmatory factor analyses and estimates of fit for the factor model per sample

Items	Standardized factor loading					
	Gs1 *(n = 508)*	Gs1 60% *(n = 305)* ^***1***^	**Gs1 40%** *(n = 203)* ^***2***^	Gs2 *(n = 279)*	RAs *(n = 121)*	ADs *(n = 70)*
**Subscale 1 – Trust in medication**						
2) I think that medication can help with my symptoms.	0.79	0.76	0.83	0.74	0.65	0.66
6) I trust that it is safe to take medication.	0.78	0.75	0.80	0.76	0.65	0.83
10) I trust in the effectiveness of medication.	0.82	0.75	0.91	0.74	0.73	0.94
12) For me the advantages of medication outweigh the disadvantages.	0.71	0.76	0.65	0.68	0.76	0.52
**Subscale 2 – Concerns about medication**						
1) I am concerned about the side effects of medication.	0.74	0.79	0.67	0.76	0.85	0.92
3) I am afraid that medication has a harmful effect on my body.	0.88	0.90	0.82	0.80	0.85	0.88
11) I am afraid of becoming addicted if I take medication for an extended period.	0.50	0.49	0.55	0.29	0.41	0.43
**Subscale 3 – Reluctance to use medication**						
4) If I have symptoms, I readily take medication for them.	-0.61	-0.64	-0.56	-0.72	-0.52	-0.71
5) I find it unnatural to take medication.	0.78	0.78	0.78	0.64	0.66	0.83
7) I only take medication if there is absolutely no other option.	0.42	0.47	0.35	0.62	0.10	0.47
8) I have no problem with taking medication if I have symptoms.	-0.77	-0.75	-0.79	-0.83	-0.78	-0.79
9) I would rather endure my symptoms than take medication.	0.80	0.79	0.83	0.71	0.76	0.72
**Indicators of model fit**						
Comparative Fit Index	0.98	-	0.97	0.98	0.98	0.98
Root Mean Square Error of Approximation [90% confidence interval]	0.10 [0.09;0.11]	-	0.12 [0.10;0.13]	0.08 [0.07;0.10]	0.08 [0.05;0.11]	0.08 [0.04;0.12]
Standardized Root Means Square Residual	0.07	-	0.09	0.07	0.09	0.10

*Note* The GAMQ was formulated and tested in Dutch. Presented here are the items from the English version obtained by forward-backward translation. ^1^ confirmatory factor analysis in training sample (*n* = 305). ^2^ confirmatory factor analysis in remaining 40% of Gs1 (*n* = 203). Gs1: General sample 1, Gs2: General sample 2, RAs: Rheumatoid arthritis sample, ADs: Atopic dermatitis sample

The Comparative Fit Index suggested an adequate fit of the factor structure for all samples, according to the classic cut-off (Table 3). The Root Mean Square Error of Approximation suggested that the fit is below adequate in all samples, however, this should be interpreted in the light of the cut-offs being suboptimal with small samples. The Standardized Root Means Square Residual indicated a close to adequate fit in all samples, being just above or below the classic cut-off in all samples, with the lowest value in ADs. Taken together, and considering the highly differential nature of the samples as well as the small sample sizes of RAs and ADs, the fit of the factor structure was deemed satisfactory across samples.

### Scoring of the GAMQ, frequency distribution of scores, and use of each item

To obtain the total score for the GAMQ, scores on the negatively worded items (items 1, 3, 5, 7, 9, 11) were reversed and the sum-score of all 12 items was calculated. This resulted in scores with a theoretical minimum of 12 and maximum of 60 with higher scores reflecting a more positive attitude. The subscales Trust and Concerns determined during factor analysis (see Table 3) contain either only positively or only negatively worded items, respectively; therefore sum scores were calculated while items were not reversed. For the subscale Reluctance, positively worded items 4 and 8 were reversed before the sum scores were calculated, with higher scores indicating greater reluctance. Visual inspection of histograms indicated that the distribution of scores for the GAMQ total and its subscales was close to normal in all samples. Generally, the distribution of the total and subscale scores covered the full or almost full theoretical range. Frequency distribution of scores for each item indicated that for most items all answering categories were used in all samples. In case a response option was not used, this favored a more positive attitude towards medication.

### Internal consistency GAMQ

Internal consistency of the GAMQ total and subscales, as indicated by Omega total (ω_t_), is displayed in Table 4. Omega total was generally good for the total scale and acceptable to good for the subscales. Omega total if each separate item is omitted is reported in Tables S3 to S6. For the total scale and subscales Trust and Reluctance no item stood out as significantly affecting the internal consistency. For the subscale Concerns, omitting item 11 (‘I am afraid of becoming addicted if I take medication for an extended period’) would increase Omega total. However, considering its content and fit in the total scale, item 11 was kept in the questionnaire.

**Table 4:** Omega total (ω_t_) as indicator of internal consistency for the GAMQ total and subscale scores for each sample

Scale	Omega			
	Gs1 *(n = 508)*	Gs2 *(n = 279)*	RAs *(n = 121)*	ADs *(n = 70)*
GAMQ Total	0.85	0.85	0.79	0.82
GAMQ Trust	0.81	0.75	0.70	0.72
GAMQ Concerns	0.71	0.63	0.73	0.76
GAMQ Reluctance	0.78	0.80	0.70	0.79

*Note* Gs1: General sample 1, Gs2: General sample 2, RAs: Rheumatoid arthritis sample, ADs: Atopic dermatitis sample. GAMQ: General Attitude towards Medication Questionnaire

As visible in Table 5, the GAMQ total correlated strongly with the Trust and Concerns subscales across samples, while only small to moderate correlations with the Reluctance subscale were observed. As can be expected, correlations with Trust were positive, while correlations with Concerns and Reluctance were negative. The subscale Trust correlated negatively with Concerns, with moderate associations in the general samples and weak or even non-significant associations in the patient samples. Associations between the Trust and Reluctance subscales were weak and generally non-significant. The subscales Concerns and Reluctance correlated weakly and positively with each other in all samples, except for a non-significant correlation in the ADs.

**Table 5:** Associations of the GAMQ with its own subscales and other indicators of attitudes towards medication

	*Gs1 (n = 508)*				*Gs2 (n = 279)*				*RAs (n = 121)*				*ADs (n = 70)*			
	Total	Trust	Concerns	Reluctance	Total	Trust	Concerns	Reluctance	Total	Trust	Concerns	Reluctance	Total	Trust	Concerns	Reluctance
**Medication attitude**																
GAMQ Total, r		**0.78** ^*^	**− 0.70** ^*^	**− 0.31** ^*^		**0.80** ^*^	**− 0.67** ^*^	− 0.19^*^		**0.71** ^*^	**− 0.66** ^*^	− 0.20^*^		**0.72** ^*^	**− 0.57** ^*^	**− 0.32** ^*^
GAMQ Trust, r			**− 0.36** ^*^	− 0.14^*^			**− 0.35** ^*^	0.00			− 0.23^*^	0.07			− 0.11	− 0.11
GAMQ Concerns, r				0.27^*^				0.20^*^				0.25^*^				0.18
GAMQ Reluctance, r																
BMQ-G General-Harm, r	**− 0.58** ^*^	**− 0.47** ^*^	**0.46** ^*^	**0.33** ^*^	**− 0.54** ^*^	**− 0.43** ^*^	**0.44** ^*^	0.28^*^	**− 0.51** ^*^	**− 0.37** ^*^	**0.47** ^*^	0.26^*^	**− 0.59** ^*^	**− 0.40** ^*^	**0.39** ^**^	**0.38** ^*^
BMQ-G General-Overuse, r	**− 0.58** ^*^	**− 0.45** ^*^	**0.46** ^*^	0.28^*^	**− 0.57** ^*^	**− 0.52** ^*^	**0.37** ^*^	0.22^*^	**− 0.50** ^*^	**− 0.37** ^*^	**0.43** ^*^	0.15	**− 0.53** ^*^	**− 0.45** ^*^	0.27^*^	0.26^*^
Medication attitude (VAS), r	**0.67** ^*^	**0.62** ^*^	**− 0.46** ^*^	− 0.23^*^	**0.69** ^*^	**0.60** ^*^	**− 0.40** ^*^	− 0.19^*^	**0.64** ^*^	**0.46** ^*^	**− 0.45** ^*^	− 0.18	**0.65** ^*^	**0.61** ^*^	− 0.28	− 0.04

*Note* Gs1: General sample 1, Gs2: General sample 2, RAs: Rheumatoid arthritis sample, ADs: Atopic dermatitis sample. GAMQ: General Attitude towards Medication Questionnaire, BMQ-G: general scale of the Beliefs about Medicines Questionnaire, VAS: visual analogue scale. ^*^ bootstrapped confidence intervals did not include 0. Medium and large effect sizes are printed in bold

### Sample comparisons on medication attitude

See Table 6 for an overview of the medication attitude scores per sample. Comparisons of the samples on the GAMQ total and subscale scores, the BMQ subscale scores for Harm and Overuse and medication attitude VAS scores showed only minimal differences between the samples. On the GAMQ total score and subscale Trust, the Gs2 scored slightly lower than the other samples (total *p* < .05, Trust *p* < .01), which did not differ from each other. On the BMQ-G subscale Harm, the ADs scored slightly lower than all other samples (*p* < .05), while the Gs2 scored slightly lower than the Gs1 (*p* = .001). On the General-Overuse subscale, the Gs1 scored significantly higher than the Gs2 (*p* < .05), the RAs, and ADs (*p* < .01); the RAs and ADs scored significantly lower than the Gs2 but did not significantly differ from each other. On medication attitude VAS scores the Gs2 scored slightly lower than the Gs1 (*p* < .01).

**Table 6:** Medication attitude for each sample – mean (SD)

	Gs1 *(n = 508)*	Gs2 *(n = 279)*	RAs *(n = 121)*	ADs *(n = 70)*	Sample comparisons
GAMQ Total	39.83 (7.01)	38.10 (7.23)	39.86 (6.23)	40.37 (6.73)	F(3,974) = 4.53, *p* = .004, η_p_^2^ = 0.01
GAMQ Trust	15.20 (2.48)	14.61 (2.50)	15.59 (2.21)	15.53 (2.29)	F(3,974) = 6.38, *p* < .001, η_p_^2^ = 0.02
GAMQ Concerns	8.76 (2.52)	9.08 (2.42)	9.11 (2.48)	8.74 (2.59)	F(3,974) = 1.38, *p* = .246, η_p_^2^ = 0.00
GAMQ Reluctance	15.10 (1.80)	15.03 (1.95)	15.03 (2.16)	15.01 (1.72)	F(3,974) = 0.12, *p* = .947, η_p_^2^ = 0.00
BMQ-G General-Harm	10.60 (2.74)	9.90 (2.62)	10.21 (2.45)	9.01 (2.66)	F(3,968) = 9.33, *p* < .001, η_p_^2^ = 0.03
BMQ-G General-Overuse	12.51 (3.00)	12.03 (3.13)	11.39 (2.68)	11.21 (3.01)	F(3,970) = 7.28, *p* < .001, η_p_^2^ = 0.02
Medication attitude VAS	63.55 (23.62)	58.58 (22.72)	63.76 (23.38)	63.34 (19.59)	F(3,949) = 3.07, *p* = .027, η_p_^2^ = 0.01

*Note* Gs1: General sample 1, Gs2: General sample 2, RAs: Rheumatoid arthritis sample, ADs: Atopic dermatitis sample. BMQ-G: general scale of the Beliefs about Medicines Questionnaire, VAS: visual analogue scale, GAMQ: General Attitude towards Medication Questionnaire

### Convergent validity: associations of the GAMQ with measures of medication attitude

Convergent validity of the GAMQ was tested by investigating the associations of the GAMQ total and subscale scores with the BMQ-G General-Harm and General-Overuse scores and the medication attitude VAS. As visible in Table 5, a more positive general medication attitude according to the GAMQ Total consistently showed strong negative correlations with the BMQ-G subscales and correlated positively and strongly with a more positive medication attitude VAS score across samples. The positive GAMQ subscale Trust consistently correlated negatively with the BMQ-G subscale scores and positively with the medication attitude VAS score. The negative GAMQ subscale Concerns was positively correlated with both BMQ-G subscale scores and negatively correlated with the medication attitude VAS score in all samples. These correlations were moderate to strong in all samples. The negative GAMQ subscale Reluctance showed weak to moderate positive correlations with the BMQ-G subscale scores, while the weak positive associations with the medication attitude VAS score were only significant in the general samples.

### Concurrent validity: associations of the GAMQ with expected medication effects

All associations of the GAMQ total and subscale scores with expected medication effects can be found in Table S7 and only statistically significant, moderate associations are reported here. Expected effectiveness of medication for pain was positively associated with the GAMQ Total in the RAs and with the subscale Trust in the Gs1 and ADs. Expected effectiveness of medication for itch was positively associated with the GAMQ Total and the subscale Trust in the ADs. Expected side effects were negatively correlated with the GAMQ Total and the subscale Trust in the ADs.

### Associations between the GAMQ and demographic and health variables

All associations of the GAMQ total and subscale scores with demographic and health variables can be found in Table S7. Only statistically significant, moderate associations are reported here. Religious or ideological affiliation was moderately associated with the GAMQ Total and the subscale Trust in the RAs. Respondents who were not affiliated with any religion or who identified as Christian had a more positive attitude towards medication and more trust than respondents affiliated with other religions. Concerning health, in the ADs, respondents who had comorbidities scored moderately higher on the subscale Trust.

## Discussion

To facilitate research into the effects of both positive and negative attitudes towards medication in general across diverse samples, we developed and evaluated the General Attitude towards Medication Questionnaire (GAMQ). We investigated the psychometric properties of the GAMQ in two general and two patient samples (rheumatoid arthritis, RAs, and atopic dermatitis, ADs). Results showed the GAMQ to consist of three subscales, which, considering classic cut-offs, generally had an adequate or close to adequate fit across samples. Furthermore, we observed the GAMQ to have good internal consistency of the total score, good convergent validity with related scales, and to be associated with expectations of pain- and itch-relieving medication, while it was not consistently or strongly associated with demographic or health-related characteristics.

Exploratory and subsequent confirmatory factor analyses indicated that the GAMQ comprises three subscales that were named Trust in medication (i.e., Trust), Concerns about medication (i.e., Concerns), and Reluctance to use medication (i.e., Reluctance). The fit of the factor structure was generally adequate or close to adequate across all samples, considering classic cut-offs. These indices may be considered relatively high given the construct we aimed to capture, as attitudes may vary within persons depending on context. Nonetheless, the suboptimal values for some of these fit indices point to the need for further attention to the factor structure in future research. Currently we cannot yet fully rule out that the subthreshold fit values could be attributed to either factor (in)stability or that they are limited by the amount of noise in the construct of interest. Also, one item (item 11) affected the internal consistency of the subscale Concerns, but was left in as it addresses the topic of fear of addiction to medication and as it did not negatively affect the internal consistency of the total scale. This topic is deemed highly relevant for this questionnaire, as it was for other scales like the Beliefs about Medicines Questionnaire (BMQ) [2] and Pain Medication Attitudes Questionnaire (PMAQ) [1], because fear of medication addiction is a concern for various medications (e.g., consider the opioid crisis [1, 45] and benzodiazepine addiction [46]). The relative consistency of the GAMQ factor structure fit across samples is not due to a lack of diversity of the investigated samples, as they differed with regard to important demographic factors (e.g., age, gender, and education) as well as health variables (chronic medical complaints, physical health-related quality of life, current pain and itch) and medication attitude (BMQ-G and GAMQ), although the latter differences were small. The internal consistency can generally be considered as good for the GAMQ total scale and acceptable for the three subscales.

Convergent validity of the GAMQ total scale and subscales was good, as indicated by the strength and direction of associations with the BMQ-G and a single item measure of medication attitude in all samples. This demonstrates the suitability of the GAMQ to measure general positive attitudes towards medication in addition to negative ones which have received more attention in the literature [2, 7–10]. Moreover, the relatively low correlations of the Reluctance subscale with the other GAMQ scores and the other medication attitude scores suggests it might measure a unique construct. Reluctance does not appear to be adequately captured in the BMQ-G scales or an assessment of general medication attitude with a single visual analogue scale. Also, it appeared less predictive of expected effectiveness and side effects of medication than the other GAMQ subscales. As the Reluctance subscale enquires about how readily patients may take medication, it could be speculated that it might be primarily predictive of treatment use, including adherence, and then indirectly of treatment outcomes. Further research into the properties of the subscales, particularly the Reluctance subscale, is required, possibly warranting further refinement of the GAMQ.

Concurrent validity of the GAMQ was investigated by examining its association with expectations about effectiveness of medication for pain and itch relief and expected side effects of medication. Overall, expected effectiveness and side effects of medication seemed to be most strongly associated with the Total score and the Trust subscale score. This provides indications for the additional value of measuring general positive attitudes to medication. It may also hint towards a link between positive attitudes toward medication and expectancy-induced placebo and nocebo effects, in line with previous research showing that positive attitudes toward medication may increase medication effectiveness [10, 13, 14]. Notably, associations of the GAMQ total and subscale scores with expected effectiveness of medication for pain were mostly stronger than for itch in the RA sample including many respondents who were experienced with taking pain medication, while the opposite was true for the AD sample including many respondents who were experienced with taking medication against itch. These findings suggest that the GAMQ total score might also be informative in predicting people’s medication-specific expectations, although further research is required to confirm this.

The GAMQ was not consistently or strongly associated with demographic or health-related characteristics. Correlations that were found (i.e., associations with religious or ideological affiliation and the presence of a (co-morbid) illness in some samples) were largely consistent with research on other measures of general medication attitudes such as the BMQ-G [47–51].

When considering the value of the GAMQ and the applicability of the current results to different samples, several limitations should be taken into account. First, the development of the GAMQ was informed by existing questionnaires assessing attitudes towards and beliefs about medication and evaluated with experts in medical and health psychology, but it did not explicitly follow a theoretical or conceptual framework, nor were the items evaluated by individuals from target populations. These approaches are highly recommendable for further scale development. Second, the GAMQ was developed and validated in Dutch. An English version of the questionnaire, obtained by forward-backward translation with the help of a certified translator and native speaker, is provided in this article. The psychometric properties of this translated version are yet to be tested. Since other questionnaires assessing general medication attitudes are valid across different languages and cultural backgrounds [e.g., 52–56], the same could be expected for the GAMQ, but this should be investigated. Similarly, one might consider to what extend the data are timebound (e.g., consider possible changes due to the COVID-19 pandemic). Third, the psychometric properties of the GAMQ were considered to be satisfactory, but further research is required and improvements might be made. In future research, attention should be paid to the factor structure, the characteristics of particularly the Reluctance subscale, and item 11 which did not have a perfect fit in the questionnaire. Also additional psychometric properties such as the temporal stability of the GAMQ scores should be investigated in future research. Additionally, item response theory may be used for validation on item level [e.g., 57]. Fourth, the completion rates of the full surveys were not high, as is commonly seen in survey research [58–60]. The length of the full surveys in the different samples may partially explain this, but other reasons such as disinterest in the topic and difficulties with answering questions cannot be excluded. Consequently, the included samples may not adequately represent the studied populations in all respects. The low completion rates also resulted in relatively small sizes of the patient samples for validation purposes. However, including the patient samples allowed for the GAMQ to be investigated in four distinct populations which differed from each other in important demographic and health characteristics and thus yielded valuable information about the usability of the GAMQ across diverse samples and generalizability of the results. Future research in other samples, particularly in patients with distinct conditions from those studied, should further inform on the generalizability of the results.

A strength of the GAMQ is its brevity (12 items), which enables its use in patient populations in which more lengthy questionnaires would be more burdensome [61]. The balance between positive and negative items along with the items clearly prompting for a personal view rather than a general statement [2] in the GAMQ should furthermore prevent inadvertent activation of overly positive or negative attitudes and resultant biases akin to placebo and nocebo effects [16, 22, 23]. As such, the GAMQ is likely suitable for use in experimental and clinical research and may have relevant advantages over commonly used instruments, such as the BMQ-G, for the assessment of general attitudes, or beliefs, about medication. It may be hypothesized that GAMQ scores predict treatment outcomes, and placebo and nocebo effects in particular, as our results show associations of GAMQ scores with expected treatment effectiveness and side effects and as previous research found similar associations [7–10, 13, 14]. GAMQ scores might also be predictive of adherence, given previously found associations with other measures of attitudes about medication [11, 12, 17]. Ultimately, the GAMQ might prove useful in clinical practice, but this in particular requires further examination.

## Conclusions

To conclude, the newly developed GAMQ is the first scale to assess not only negative but also positive attitudes towards medication in general, providing indicators of Trust, Concerns, and Reluctance regarding medication. The GAMQ showed satisfactory psychometric properties in terms of the factor structure, internal consistency, and convergent as well as concurrent validity in the four investigated general and patient samples. Therefore, it may be suitable for assessing general medication attitudes, both negative and positive, in a variety of research settings and populations. The balanced nature and brevity of the GAMQ render it a promising novel tool for investigating predictors of medication outcomes and adherence, as well as placebo and nocebo effects.

## Electronic supplementary material

Below is the link to the electronic supplementary material.

Supplementary Material 1

## Data Availability

Study materials, data, analyses scripts, and output are deposited in the online archiving system DataverseNL at 10.34894/HGTM1Y. Due to privacy issues, the data are not shared publicly, but will be made available to individuals upon reasonable request.

## Abbreviations

GAMQ: General Attitude towards Medication Questionnaire
BMQ-G: Beliefs about Medicines Questionnaire-General
RAs: Rheumatoid arthritis sample
ADs: Atopic dermatitis sample
Gs1: General sample 1
Gs2: General sample 2
VAS: Visual analogue scale
SF12: Short Form-12
PCS: Physical health composite score of the SF12
MCS: Mental health composite score of the SF12
DAS44: Disease Activity Score 44
POEM: Patient Oriented Eczema Measure
ANOVA: Analyses of variance

## References

[1] McCracken LM, Hoskins J, Eccleston C. Concerns about medication and medication use in chronic pain. J Pain. 2006;7(10):726–34. 10.1016/j.jpain.2006.02.014. 17018333 10.1016/j.jpain.2006.02.014

[2] Horne R, Weinman J, Hankins M. The beliefs about medicines questionnaire: the development and evaluation of a new method for assessing the cognitive representation of medication. Psychol Health. 1999;14(1):1–24. 10.1080/08870449908407311.

[3] Kamping S, Müller M, Klinger R, Schmitz J, Flor H. Analgesics in Chronic Back Pain. Z Psychol. 2014;222(3):179–85. 10.1027/2151-2604/a000182.

[4] Hogan TP, Awad AG, Eastwood R. A self-report scale predictive of drug compliance in schizophrenics: reliability and discriminative validity. Psychol Med. 1983;13(1):177–83. 10.1017/s0033291700050182. 6133297 10.1017/s0033291700050182

[5] WHO WHO. Adherence to Long-term Therapies: Evidence for Action. Geneva, Switzerland2003. pp. 1–198.

[6] Manaï M, van Middendorp H, Veldhuijzen DS, Huizinga TW, Evers AW. How to prevent, minimize, or extinguish nocebo effects in pain: a narrative review on mechanisms, predictors, and interventions. Pain Rep. 2019;4(3):e699. 31583340 10.1097/PR9.0000000000000699 PMC6749907

[7] Heller MK, Chapman SC, Horne R. Beliefs about medication predict the misattribution of a common symptom as a medication side effect–evidence from an analogue online study. J Psychosom Res. 2015;79(6):519–29. 10.1016/j.jpsychores.2015.10.003. 26519128 10.1016/j.jpsychores.2015.10.003

[8] Nestoriuc Y, Orav EJ, Liang MH, Horne R, Barsky AJ. Prediction of nonspecific side effects in rheumatoid arthritis patients by beliefs about medicines. Arthritis Care Res. 2010;62(6):791–9. 10.1002/acr.20160. 10.1002/acr.20160 PMC2940323 20191574

[9] Heller MK, Chapman SCE, Horne R. Beliefs about Medicines Predict Side-effects of Placebo Modafinil. Ann Behav Med. 2022;56(10):989–1001. 10.1093/abm/kaab112. 35512392 10.1093/abm/kaab112

[10] Webster RK, Weinman J, Rubin GJ. Medicine-related beliefs predict attribution of symptoms to a sham medicine: a prospective study. Br J Health Psychol. 2018;23(2):436–54. 10.1111/bjhp.12298. 29405507 10.1111/bjhp.12298 PMC5900880

[11] Thorneloe RJ, Griffiths CEM, Emsley R, Ashcroft DM, Cordingley L. Intentional and unintentional medication non-adherence in Psoriasis: the role of patients’ medication beliefs and habit strength. J Invest Dermatol. 2018;138(4):785–94. 10.1016/j.jid.2017.11.015. 29183731 10.1016/j.jid.2017.11.015 PMC5869950

[12] Horne R, Chapman SCE, Parham R, Freemantle N, Forbes A, Cooper V. Understanding patients’ adherence-related beliefs about Medicines prescribed for long-term conditions: a Meta-Analytic Review of the necessity-concerns Framework. PLoS ONE. 2013;8(12):e80633. 10.1371/journal.pone.0080633. 24312488 10.1371/journal.pone.0080633 PMC3846635

[13] Watkinson A, Chapman SCE, Horne R. Beliefs about Pharmaceutical Medicines and natural remedies explain individual variation in Placebo Analgesia. J Pain. 2017;18(8):908–22. 10.1016/j.jpain.2017.02.435. 28279704 10.1016/j.jpain.2017.02.435

[14] MacKrill K, Petrie KJ. What is associated with increased side effects and lower perceived efficacy following switching to a generic medicine? A New Zealand cross-sectional patient survey. BMJ Open. 2018;8(10):2018–023667. 10.1136/bmjopen-2018-023667. 10.1136/bmjopen-2018-023667 PMC6196872 30341138

[15] Vase L, Petersen GL, Riley JL 3rd, Price DD. Factors contributing to large analgesic effects in placebo mechanism studies conducted between 2002 and 2007. Pain. 2009;145(1–2):36–44. 10.1016/j.pain.2009.04.008. 10.1016/j.pain.2009.04.008 19559529

[16] Blythe JS, Thomaidou MA, Peerdeman KJ, van Laarhoven AIM, van Schothorst MME, Veldhuijzen DS, Evers AWM. Placebo effects on cutaneous pain and itch: a systematic review and meta-analysis of experimental results and methodology. Pain. 2023;164(6):1181–99. 10.1097/j.pain.0000000000002820. 36718994 10.1097/j.pain.0000000000002820 PMC10184563

[17] El Abdellati K, De Picker L, Morrens M. Antipsychotic treatment failure: a systematic review on risk factors and interventions for treatment adherence in psychosis. Front Neurosci. 2020;14:531763. 10.3389/fnins.2020.531763. 33162877 10.3389/fnins.2020.531763 PMC7584050

[18] Cacioppo JT, Berntson GG. Relationship between attitudes and Evaluative Space - a Critical-Review, with emphasis on the separability of positive and negative substrates. Psychol Bull. 1994;115(3):401–23. 10.1037/0033-2909.115.3.401.

[19] Diener E, Emmons RA. The independence of positive and negative affect. J Pers Soc Psychol. 1984;47(5):1105–17. 10.1037//0022-3514.47.5.1105. 10.1037//0022-3514.47.5.1105 6520704

[20] Horne R, Faasse K, Cooper V, Diefenbach MA, Leventhal H, Leventhal E, Petrie KJ. The perceived sensitivity to medicines (PSM) scale: an evaluation of validity and reliability. Br J Health Psychol. 2013;18(1):18–30. 10.1111/j.2044-8287.2012.02071.x. 22524270 10.1111/j.2044-8287.2012.02071.x

[21] Nielsen RE, Lindstrom E, Nielsen J, Levander S. DAI-10 is as good as DAI-30 in schizophrenia. Eur Neuropsychopharmacology: J Eur Coll Neuropsychopharmacol. 2012;22(10):747–50. 10.1016/j.euroneuro.2012.02.008. 10.1016/j.euroneuro.2012.02.008 22440974

[22] Peerdeman KJ, van Laarhoven AI, Keij SM, Vase L, Rovers MM, Peters ML, Evers AW. Relieving patients’ pain with expectation interventions: a meta-analysis. Pain. 2016;157(6):1179–91. 10.1097/j.pain.0000000000000540. 26945235 10.1097/j.pain.0000000000000540

[23] Thomaidou MA, Blythe JS, Peerdeman KJ, van Laarhoven AIM, Van Schothorst MME, Veldhuijzen DS, Evers AWM. Learned Nocebo effects on cutaneous sensations of Pain and Itch: a systematic review and Meta-analysis of experimental behavioral studies on healthy humans. Psychosom Med. 2023;85(4):308–21. 10.1097/psy.0000000000001194. 36961347 10.1097/PSY.0000000000001194 PMC10171297

[24] Wolters F, Peerdeman KJ, Evers AW. Placebo and nocebo effects across symptoms: from pain to fatigue, dyspnea, nausea, and itch. Front Psychiatry. 2019;10:459431. 10.3389/fpsyt.2019.00470 PMC6614509 31312148

[25] Peerdeman KJ, Tekampe J, van Laarhoven AIM, van Middendorp H, Rippe RCA, Peters ML, Evers AWM. Expectations about the effectiveness of pain- and itch-relieving medication administered via different routes. Eur J Pain. 2018;22(4):774–83. 10.1002/ejp.1163. 29266544 10.1002/ejp.1163 PMC5873387

[26] Floridou GA, Peerdeman KJ, Schaefer R. Individual Differences in Mental Imagery in different modalities and levels of intentionality. Mem Cogn. 2022;50:29–44. 10.3758/s13421-021-01209-7. 10.3758/s13421-021-01209-7 PMC8763825 34462893

[27] Cohen J. A power primer. Psychol Bull. 1992;112(1):155–9. 10.1037//0033-2909.112.1.155. 10.1037//0033-2909.112.1.155 19565683

[28] Kyriazos TA. Applied Psychometrics: sample size and Sample Power Considerations in Factor Analysis (EFA, CFA) and SEM in General. Psychology. 2018;9:2207–30. 10.4236/psych.2018.98126.

[29] de Rooy DP, van der Linden MP, Knevel R, Huizinga TW, van der Helm-van Mil AH. Predicting arthritis outcomes–what can be learned from the Leiden. Early Arthritis Clinic? Rheumatol. 2011;50(1):93–100. 10.1093/rheumatology/keq230. 10.1093/rheumatology/keq230 20639266

[30] Arnett FC, Edworthy SM, Bloch DA, McShane DJ, Fries JF, Cooper NS, et al. The American Rheumatism Association 1987 revised criteria for the classification of rheumatoid arthritis. Arthritis Rheum. 1988;31(3):315–24. 10.1002/art.1780310302. 3358796 10.1002/art.1780310302

[31] Tsianou K, Giannakeas N, Tsipouras MG, Tzallas AT, Christodoulou DK, Bureš J, et al. Accessing patient views about medication in chronic conditions using the beliefs about Medicine Questionnaire (BMQ): a review study. J Drug Res Dev. 2017;3. 10.16966/2470-1009.130.

[32] Mols F, Pelle AJ, Kupper N. Normative data of the SF-12 health survey with validation using postmyocardial infarction patients in the Dutch population. Qual life Research: Int J Qual life Aspects Treat care Rehabilitation. 2009;18(4):403–14. 10.1007/s11136-009-9455-5. 10.1007/s11136-009-9455-5 19242822

[33] van Riel PL, Renskers L. The Disease activity score (DAS) and the Disease Activity score using 28 joint counts (DAS28) in the management of rheumatoid arthritis. Clin Exp Rheumatol. 2016;34(5 Suppl 101):S40–4. 27762189

[34] Charman CR, Venn AJ, Williams HC. The patient-oriented eczema measure: development and initial validation of a new tool for measuring atopic eczema severity from the patients’ perspective. Arch Dermatol. 2004;140(12):1513–9. 10.1001/archderm.140.12.1513. 15611432 10.1001/archderm.140.12.1513

[35] Fransen J, van Riel PL. The Disease activity score and the EULAR response criteria. Clin Exp Rheumatol. 2005;23(5 Suppl 39):S93–9. 16273792

[36] Spuls PI, Gerbens LAA, Simpson E, Apfelbacher CJ, Chalmers JR, Thomas KS, et al. Patient-oriented Eczema measure (POEM), a core instrument to measure symptoms in clinical trials: a Harmonising Outcome measures for Eczema (HOME) statement. Brit J Dermatol. 2017;176(4):979–84. 10.1111/bjd.15179. 27858989 10.1111/bjd.15179

[37] Cohen J. Statistical power analysis for the behavioral sciences. Hillsdale, N.J.: L. Erlbaum Associates; 1988.

[38] Widaman KF, Reise SP. Exploring the measurement invariance of psychological instruments: applications in the substance use domain. American Psychological Association; 1997.

[39] Meredith W. Measurement invariance, factor analysis and factorial invariance. Psychometrika. 1993;58(4):525–43. 10.1007/bf02294825.

[40] Hu L-T, Bentler PM. Fit indices in Covariance structure modeling: sensitivity to Underparameterized Model Misspecification. Psychol Methods. 1998;3(4):424–53. 10.1037/1082-989x.3.4.424.

[41] Xia Y, Yang Y, RMSEA, CFI. TLI in structural equation modeling with ordered categorical data: the story they tell depends on the estimation methods. Behav Res Methods. 2019;51(1):409–28. 10.3758/s13428-018-1055-2. 29869222 10.3758/s13428-018-1055-2

[42] Barrett P. Structural equation modelling: adjudging model fit. Pers Indiv Differ. 2007;42(5):815–24. 10.1016/j.paid.2006.09.018.

[43] Cronbach LJ. Coefficient alpha and the internal structure of tests. Psychometrika. 1951;16(3):297–334. 10.1007/bf02310555.

[44] Symmons DPM. Epidemiology of rheumatoid arthritis: determinants of onset, persistence and outcome. Best Pract Res Clin Rheumatol. 2002;16(5):707–22. 10.1053/berh.2002.0257. 12473269 10.1053/berh.2002.0257

[45] Graczyk M, Borkowska A, Krajnik M. Why patients are afraid of opioid analgesics: a study on opioid perception in patients with chronic pain. Pol Arch Intern Med. 2018;128(2):89–97. 10.20452/pamw.4167. 29240048 10.20452/pamw.4167

[46] Longo LP, Johnson B, Addiction. Part I. benzodiazepines–side effects, abuse risk and alternatives. Am Fam Physician. 2000;61(7):2121–8. 10779253

[47] Koster ES, Heerdink ER, de Vries TW, Bouvy ML. Attitudes towards medication use in a general population of adolescents. Eur J Pediatrics. 2014;173(4):483–8. 10.1007/s00431-013-2211-4. 10.1007/s00431-013-2211-4 24221610

[48] Horne R, Graupner L, Frost S, Weinman J, Wright SM, Hankins M. Medicine in a multi-cultural society: the effect of cultural background on beliefs about medications. Soc Sci Med. 2004;59(6):1307–13. 10.1016/j.socscimed.2004.01.009. 10.1016/j.socscimed.2004.01.009 15210101

[49] Drangsholt SH, Cappelen UW, von der Lippe N, Hoieggen A, Os I, Brekke FB. Beliefs about medicines in dialysis patients and after renal transplantation. Hemodial Int Int Symp Home Hemodial. 2019;23(1):117–25. 10.1111/hdi.12717. 10.1111/hdi.12717 30714322

[50] Kumar K, Gordon C, Toescu V, Buckley CD, Horne R, Nightingale PG, Raza K. Beliefs about medicines in patients with rheumatoid arthritis and systemic lupus erythematosus: a comparison between patients of south Asian and white British origin. Rheumatology (Oxford). 2008;47(5):690–7. 10.1093/rheumatology/ken050. 10.1093/rheumatology/ken050 18375972

[51] Mardby AC, Akerlind I, Jorgensen T. Beliefs about medicines and self-reported adherence among pharmacy clients. Patient Educ Couns. 2007;69(1–3):158–64. 10.1016/j.pec.2007.08.011. 17913439 10.1016/j.pec.2007.08.011

[52] Nie B, Chapman SCE, Chen Z, Wang X, Wei L. Utilization of the beliefs about medicine questionnaire and prediction of medication adherence in China: a systematic review and meta-analysis. J Psychosom Res. 2019;122:54–68. 10.1016/j.jpsychores.2019.03.184. 31006535 10.1016/j.jpsychores.2019.03.184

[53] Tan CS, Hassali MA, Neoh CF, Saleem F, Horne R. Cultural adaptation and linguistic validation of the beliefs about Medicines Questionnaire in Malaysia. Value in health regional issues. 2018;15:161–8. 10.1016/j.vhri.2017.12.010 10.1016/j.vhri.2017.12.010 29730249

[54] Cinar M, Cinar FI, Acikel C, Yilmaz S, Cakar M, Horne R, Simsek I. Reliability and validity of the Turkish translation of the beliefs about medicines questionnaire (BMQ-T) in patients with Behcet’s disease. Clin Exp Rheumatol. 2016;34(6 Suppl 102):S46–51. 27191774

[55] Tibaldi G, Clatworthy J, Torchio E, Argentero P, Munizza C, Horne R. The utility of the necessity—concerns Framework in explaining treatment non-adherence in four chronic illness groups in Italy. Chronic Illn. 2009;5(2):129–33. 10.1177/1742395309102888. 19474235 10.1177/1742395309102888

[56] Mahler C, Hermann K, Horne R, Jank S, Haefeli WE, Szecsenyi J. Patients’ beliefs about Medicines in a primary care setting in Germany. J Eval Clin Pract. 2012;18(2):409–13. 10.1111/j.1365-2753.2010.01589.x. 21087373 10.1111/j.1365-2753.2010.01589.x

[57] Nguyen TH, Han HR, Kim MT, Chan KS. An introduction to item response theory for patient-reported outcome measurement. Patient. 2014;7(1):23–35. 10.1007/s40271-013-0041-0. 24403095 10.1007/s40271-013-0041-0 PMC4520411

[58] Burgard T, Bošnjak M, Wedderhoff N. Response rates in online surveys with affective disorder participants: A meta-analysis of study design and time effects between 2008 and 2019. Zeitschrift für Psychologie. 228(1):14–24. 10.1027/2151-2604/a000394.

[59] Holtom B, Baruch Y, Aguinis H, Ballinger A. Survey response rates: Trends and a validity assessment framework. Hum Relat. 2022;75(8):1560–84.

[60] Wu M-J, Zhao K, Fils-Aime F. Response rates of online surveys in published research: a meta-analysis. Computers Hum Behav Rep. 2022;7:100206.

[61] Rolstad S, Adler J, Ryden A. Response burden and questionnaire length: is shorter better? A review and Meta-analysis. Value Health. 2011;14(8):1101–8. 10.1016/j.jval.2011.06.003. 22152180 10.1016/j.jval.2011.06.003

