# Supplementary material for: Development and validation of the General attitude towards Medication Questionnaire (GAMQ)
Source: BMC Psychol. 2024 Nov 7;12:632. doi: 10.1186/s40359-024-02108-7 (PMC11542261; doi:10.1186/s40359-024-02108-7)
Supplement: Supplementary file 1 — Supplementary Material 1 [file 40359_2024_2108_MOESM1_ESM.docx]

# Supporting information

Development and validation of the General Attitude towards Medication Questionnaire (GAMQ)

Peerdeman & Tekampe et al.

# Table S1. Overview of questionnaires per sample.

|  | **Gs1** | **Gs2** | **RAs** | **ADs** |
| --- | --- | --- | --- | --- |
| **Attitude towards medication** | |  |  |  |
| GAMQ | x | x | x | x |
| BMQ-G | x | x | x | x |
| Medication attitude VAS | x | x | x | x |
| **Health-related characteristics** | |  |  |  |
| SF-12 PCS | x |  | x | x |
| SF-12 MCS | x |  | x | x |
| POEM |  |  |  | x |
| DAS44 |  |  | x |  |
| Current pain intensity | x | x | x | x |
| Current itch intensity | x |  | x | x |
| In treatment for long-lasting medical or psychological complaints or diseases (other than RA for RAs and AD for ADs) | x | x | x | x |
| **Expectancies regarding medication** | x |  | x | x |

Note. Gs1: General sample 1, Gs2: General sample 2, RAs: Rheumatoid Arthritis sample, ADs: Atopic dermatitis sample, GAMQ: General Attitude towards Medication Questionnaire, BMQ-G: General scale of the Beliefs about Medicines Questionnaire, VAS: Visual Analogue Scale, SF-12: Short Form 12, PCS: Physical health Composite, MCS: Mental health Composite, POEM: Patient Oriented Eczema Measure, DAS44: Disease Activity Score-44.

# Table S2. Original Dutch version of the General Attitude toward Medication Questionnaire (Attitude ten opzichte van Medicijnen Vragenlijst)

| Wij willen u nu een aantal vragen stellen over hoe u denkt over medicijnen in het algemeen.  Wij zijn geïnteresseerd in uw mening over medicijnen die u op doktersvoorschrift gebruikt. *  Wilt u aangeven in hoeverre u het eens of oneens bent met onderstaande uitspraken?  Er zijn geen goede of foute antwoorden. Wij zijn benieuwd naar uw mening. | | | | | |
| --- | --- | --- | --- | --- | --- |
|  | helemaal mee oneens | mee oneens | geen duidelijke mening | mee eens | helemaal mee eens |
| 1. Ik ben bezorgd over de bijwerkingen van medicijnen |  |  |  |  |  |
| 1. Ik denk dat medicijnen mij goed kunnen helpen tegen klachten |  |  |  |  |  |
| 1. Ik ben bang dat medicijnen schadelijk zijn voor mijn lichaam |  |  |  |  |  |
| 1. Als ik klachten heb, neem ik daar gemakkelijk medicijnen voor |  |  |  |  |  |
| 1. Ik vind het onnatuurlijk om medicijnen te gebruiken |  |  |  |  |  |
| 1. Ik vertrouw er op dat het veilig is om medicijnen te gebruiken |  |  |  |  |  |
| 1. Ik gebruik medicijnen alleen als er echt geen andere mogelijkheid is |  |  |  |  |  |
| 1. Ik heb er geen moeite mee om medicijnen te gebruiken als ik klachten heb |  |  |  |  |  |
| 1. Ik onderga mijn klachten liever dan dat ik medicijnen gebruik |  |  |  |  |  |
| 1. Ik vertrouw op de werkzaamheid van medicijnen |  |  |  |  |  |
| 1. Ik ben bang verslaafd te raken als ik medicijnen langdurig gebruik |  |  |  |  |  |
| 1. Voor mij wegen de voordelen van medicijnen op tegen de nadelen |  |  |  |  |  |

* Post hoc we realized this sentence could be misinterpreted as indicating that we are interested in their attitudes towards medications that have been specifically prescribed to them by their own doctor, while were are interested in all medications that are available upon prescription by any doctor. We therefore recommend to adjust it in future research to the following: “Wij zijn geïnteresseerd in uw mening over medicijnen die op doktersvoorschrift beschikbaar zijn.”

# Table S3. Omega total (ω_t_) of the GAMQ total scale if item omitted.

| **Item** | **Gs1**  *(n = 508)* | **Gs2**  *(n = 279)* | **RAs**  *(n = 121)* | **ADs**  *(n = 70)* |
| --- | --- | --- | --- | --- |
| 1) I am concerned about the side effects of medication. *(rev)* | .85 | .84 | .78 | .81 |
| 2) I think that medication can help with my symptoms. | .84 | .83 | .77 | .82 |
| 3) I am afraid that medication has a harmful effect on my body. *(rev)* | .84 | .84 | .77 | .81 |
| 4) If I have symptoms, I readily take medication for them. | .85 | .83 | .78 | .80 |
| 5) I find it unnatural to take medication. *(rev)* | .83 | .83 | .76 | .79 |
| 6) I trust that it is safe to take medication. | .84 | .83 | .77 | .79 |
| 7) I only take medication if there is absolutely no other option. *(rev)* | .85 | .83 | **.80** | .81 |
| 8) I have no problem with taking medication if I have symptoms. | .84 | .82 | .77 | .79 |
| 9) I would rather endure my symptoms than take medication. *(rev)* | .83 | .83 | .76 | .80 |
| 10) I trust in the effectiveness of medication. | .84 | .83 | .78 | .80 |
| 11) I am afraid of becoming addicted if I take medication for an extended period. *(rev)* | .85 | .85 | .79 | **.83** |
| 12) For me the advantages of medication outweigh the disadvantages. | .84 | .83 | .77 | .82 |
| **Total scale** | **.85** | **.85** | **.79** | **.82** |

*Note*: Gs1: General sample 1, Gs2: General sample 2, RAs: Rheumatoid Arthritis sample, ADs: Atopic dermatitis sample. (rev) indicates items for which scores were reversed.

# Table S4. Omega total (ω_t_) of the GAMQ subscale Trust if item omitted.

| **Item** | **Gs1**  *(n = 508)* | **Gs2**  *(n = 279)* | **RAs**  *(n = 121)* | **ADs**  *(n = 70)* |
| --- | --- | --- | --- | --- |
| 2) I think that medication can help with my symptoms. | .75 | .70 | .65 | .69 |
| 6) I trust that it is safe to take medication. | .78 | .70 | .67 | .62 |
| 10) I trust in the effectiveness of medication. | .73 | .67 | .61 | .55 |
| 12) For me the advantages of medication outweigh the disadvantages. | .80 | .73 | .63 | **.76** |
| **Total scale** | **.81** | **.75** | **.70** | **.72** |

*Note*: Gs1: General sample 1, Gs2: General sample 2, RAs: Rheumatoid Arthritis sample, ADs: Atopic dermatitis sample.

# Table S5. Omega total (ω_t_) of the GAMQ subscale Concerns if item omitted.

| **Item** | **Gs1**  *(n = 508)* | **Gs2**  *(n = 279)* | **RAs**  *(n = 121)* | **ADs**  *(n = 70)* |
| --- | --- | --- | --- | --- |
| 1) I am concerned about the side effects of medication. | .56 | .38 | .35 | .45 |
| 3) I am afraid that medication has a harmful effect on my body. | .48 | .41 | .50 | .48 |
| 11) I am afraid of becoming addicted if I take medication for an extended period. | **.74** | **.68** | **.81** | **.85** |
| **Total scale** | **.71** | **.63** | **.73** | **.76** |

*Note*: Gs1: General sample 1, Gs2: General sample 2, RAs: Rheumatoid Arthritis sample, ADs: Atopic dermatitis sample.

# Table S6. Omega total (ω_t_) of the GAMQ subscale Reluctance if item omitted.

| **Item** | **Gs1**  *(n = 508)* | **Gs2**  *(n = 279)* | **RAs**  *(n = 121)* | **ADs**  *(n = 70)* |
| --- | --- | --- | --- | --- |
| 4) If I have symptoms, I readily take medication for them. *(rev)* | .75 | .75 | .66 | .75 |
| 5) I find it unnatural to take medication. | .73 | .78 | .64 | .74 |
| 7) I only take medication if there is absolutely no other option. | .78 | .77 | **.72** | **.80** |
| 8) I have no problem with taking medication if I have symptoms. *(rev)* | .74 | .74 | .62 | .75 |
| 9) I would rather endure my symptoms than take medication. | .72 | .75 | .59 | .73 |
| **Total scale** | **.78** | **.80** | **.70** | **.79** |

*Note*: Gs1: General sample 1, Gs2: General sample 2, RAs: Rheumatoid Arthritis sample, ADs: Atopic dermatitis sample.

# Table S7. Correlations of the GAMQ with demographic characteristics, health variables, and expected medication effects.

|  | **Gs1** *(n = 508)* | | | | **Gs2** *(n = 279)* | | | | **RAs** *(n = 121)* | | | | **ADs** *(n = 70)* | | | |
| --- | --- | --- | --- | --- | --- | --- | --- | --- | --- | --- | --- | --- | --- | --- | --- | --- |
|  | **Total** | **Trust** | **Concerns** | **Reluctance** | **Total** | **Trust** | **Concerns** | **Reluctance** | **Total** | **Trust** | **Concerns** | **Reluctance** | **Total** | **Trust** | **Concerns** | **Reluctance** |
| **Demographic characteristics** |  |  |  |  |  |  |  |  |  |  |  |  |  |  |  |  |
| Age (years), r | .04 | .12^*^ | .00 | .00 | .02 | .0 | - .01 | -.03 | .02 | -.10 | .00 | .06 | -.03 | .02 | .06 | .14 |
| Sex, η_p_^2^ | .02^**^ | .01^*^ | .03^***^ | .01^*^ | .01 | .00 | .01 | .00 | .03 | .02 | .00 | .00 | .00 | .01 | .04 | .01 |
| Men, mean (SD) | 40.94 (6.47) | 15.48 (2.45) | 8.28  (2.36) | 14.92  (1.78) | 38.78 (7.55) | 14.74 (2.66) | 8.85  (2.51) | 15.11  (2.03) | 41.52 (6.53) | 16.10 (2.41) | 9.00  (2.53) | 14.87  (2.38) | 39.97 (7.64) | 15.71 (2.52) | 9.35  (2.78) | 15.16  (1.68) |
| Women, mean (SD) | 38.79 (7.34) | 14.94 (2.49) | 9.21 (2.59) | 15.27 (1.80) | 37.50 (6.92) | 14.50 (2.35) | 9.28 (2.32) | 14.96 (1.88) | 39.29 (6.05) | 15.41 (2.12) | 9.14 (2.48) | 15.09 (2.10) | 40.69 (6.00) | 15.38 (2.11) | 8.26 (2.36) | 14.90 (1.77) |
| Educational level, η_p_^2^ | .01 | .01 | .00 | .00 | .00 | .00 | .00 | .00 | .01 | .01 | .00 | .00 | .02 | .02 | .04 | .01 |
| Secondary, mean (SD) | 40.23  (6.96) | 15.35 (2.48) | 8.68 (2.48) | 15.16 (1.75) | 37.46 (7.20) | 14.44 (2.65) | 9.24 (2.55) | 15.04 (2.15) | 40.33 (6.05) | 15.72 (2.15) | 9.14 (2.60) | 15.14 (2.38) | 39.38 (7.42) | 15.22 (2.42) | 9.28 (2.99) | 15.19 (1.45) |
| Tertiary, mean (SD) | 39.22 (7.05) | 14.96 (2.47) | 8.88 (2.59) | 15.01 (1.87) | 38.35 (7.19) | 14.70 (2.42) | 8.99 (2.35) | 15.02 (1.84) | 38.90 (6.54) | 15.32 (2.33) | 9.05 (2.26) | 14.83 (1.65) | 41.21 (6.06) | 15.79 (2.17) | 8.29 (2.14) | 14.87 (1.93) |
| Nationality, η_p_^2^ | .01 | .00 | .01 | .01 | .00 | .00 | .01 | .01 | .02 | .03 | .02 | .00 | .03 | .03 | .03 | .01 |
| Dutch, mean (SD) | 39.86 (6.97) | 15.22 (2.47) | 8.75 (2.50) | 15.12 (1.80) | 38.14 (7.24) | 14.65 (2.51) | 9.12 (2.41) | 15.06 (1.93) | 39.96 (6.21) | 15.62 (2.21) | 9.12 (2.50) | 15.04 (2.17) | 40.52 (6.73) | 15.58 (2.30) | 8.66 (2.56) | 14.97 (1.75) |
| Other, mean (SD) | 35.17 (9.83) | 14.00 (3.74) | 10.17 (3.66) | 14.33 (1.37) | 36.30 (6.78) | 14.10 (2.13) | 8.80 (2.53) | 14.60 (2.55) | 35.67 (4.93) | 13.33 (1.53) | 8.00 (1.73) | 15.00 (2.65) | 40.50 (4.95) | 15.50 (0.71) | 11.00 (4.24) | 16.00 (0.00) |
| Multiple, mean (SD) | 42.80 (5.40) | 15.20 (2.05) | 7.60 (2.79) | 13.60 (1.14) | 39.33 (8.21) | 13.67 (2.73) | 7.83 (2.56) | 14.17 (1.94) | 33.00 (n=1) | 17.00 (n=1) | 12.00 (n=1) | 16.00 (n=1) | 30.00 (n=1) | 12.00 (n=1) | 10.00 (n=1) | 16.00 (n=1) |
| Religious or ideological affiliation, η_p_^2^ | .01 | .00 | .01 | .03** | .00 | .00 | .00 | .01 | **.07*** | **.08**** | .02 | .01 | **.06** | .00 | **.07** | .02 |
| None, mean (SD) | 40.19 (6.79) | 15.26 (2.39) | 8.67 (2.43) | 14.93 (1.71) | 38.11 (7.42) | 14.64 (2.58) | 9.03 (2.52) | 15.12 (1.95) | 39.69 (5.46) | 15.63 (2.12) | 9.34 (2.25) | 15.27 (2.03) | 41.21 (6.49) | 15.69 (2.21) | 8.31 (2.51) | 15.21 (2.08) |
| Christian, mean (SD) | 39.61 (7.18) | 15.21 (2.46) | 8.78 (2.55) | 15.48 (1.77) | 38.08 (6.05) | 14.57 (2.26) | 9.26 (1.86) | 14.77 (1.89) | 40.49 (6.57) | 15.75 (2.04) | 8.79 (2.74) | 14.79 (2.35) | 40.51 (6.83) | 15.57 (2.36) | 8.84 (2.62) | 14.86 (1.44) |
| Other, mean (SD) | 37.63 (7.84) | 14.60 (3.37) | 9.53 (3.10) | 14.57 (2.37) | 38.00 (10.69) | 14.20 (2.62) | 8.90 (3.48) | 14.90 (2.33) | 31.25 (6.18) | 12.25 (3.77) | 10.50 (1.73) | 15.50 (0.58) | 33.00 (4.00) | 15.00 (1.00) | 11.67 (2.08) | 15.67 (1.15) |
| Marital status, η_p_^2^ | .00 | .00 | .00 | .00 | .00 | .01 | .00 | .00 | .00 | .01 | .01 | .00 | .04 | .02 | .03 | .02 |
| Single, mean (SD) | 39.55 (6.61) | 15.10 (2.34) | 8.88 (2.62) | 15.02 (1.95) | 37.51 (7.89) | 14.30 (2.74) | 9.01 (2.64) | 15.09 (2.14) | 40.23 (6.71) | 15.91 (1.88) | 8.55 (3.05) | 14.77 (2.00) | 42.45 (5.96) | 16.00 (2.25) | 8.05 (2.39) | 14.60 (2.01) |
| In relationship, mean (SD) | 39.98 (7.21) | 15.26 (2.55) | 8.70 (2.47) | 15.14 (1.71) | 38.38 (6.91) | 14.75 (2.37) | 9.11 (2.31) | 15.00 (1.86) | 39.50 (5.85) | 15.45 (2.26) | 9.30 (2.28) | 15.15 (2.21) | 39.54 (6.90) | 15.34 (2.30) | 9.02 (2.64) | 15.18 (1.59) |

| **Health variables** |  |  |  |  |  |  |  |  |  |  |  |  |  |  |  |  |
| --- | --- | --- | --- | --- | --- | --- | --- | --- | --- | --- | --- | --- | --- | --- | --- | --- |
| long-lasting medical or psychological complaints ^1^, η_p_^2^ | .02^**^ | .03^***^ | .01 | .00 | .00 | .01 | .01 | .00 | .00 | .00 | .00 | .00 | .05 | **.11^**^** | .00 | .01 |
| No, mean (SD) | 39.10 (7.06) | 14.83 (2.61) | 8.60 (2.40) | 15.14 (1.85) | 37.88 (7.68) | 14.49 (2.60) | 8.92 (2.47) | 14.97 (1.91) | 39.72(6.52) | 15.57 (2.49) | 9.15 (2.44) | 15.03 (1.70) | 39.08 (7.27) | 14.87 (2.28) | 8.82 (2.49) | 15.13 (1.67) |
| Yes, mean (SD) | 40.81 (6.83) | 15.70 (2.21) | 8.97 (2.67) | 15.05 (1.72) | 38.58 (6.15) | 14.87 (2.25) | 9.42 (2.28) | 15.15 (2.04) | 40.31 (6.00) | 15.63 (1.87) | 9.02 (2.58) | 14.93 (2.57) | 42.00 (5.69) | 16.35 (2.04) | 8.65 (2.75) | 14.87 (1.80) |
| SF-12 PCS, r | .00 | - .08 | - .18^*^ | -.04 | n.a. | n.a. | n.a. | n.a. | .20^*^ | .17 | -.17 | -.21 | -.15 | -.10 | -.03 | .07 |
| SF-12 MCS, r | .06 | .04 | - .23^*^ | .02 | n.a. | n.a. | n.a. | n.a. | .14 | .01 | -.29^*^ | -.25* | -.06 | -.04 | -.10 | .17 |
| DAS44, r | n.a. | n.a. | n.a. | n.a. | n.a. | n.a. | n.a. | n.a. | -.24^*^ | -.25* | .16 | .21^*^ | n.a. | n.a. | n.a. | n.a. |
| POEM, r | n.a. | n.a. | n.a. | n.a. | n.a. | n.a. | n.a. | n.a. | n.a. | n.a. | n.a. | n.a. | .01 | .06 | .10 | .01 |
| Current pain intensity, r | -.02 | .05 | .14^*^ | .02 | .00 | -.01 | .09 | .12 | -.07 | -.07 | .08 | .10 | .08 | .09 | .20^*^ | -.16 |
| Current itch intensity, r | .02 | .00 | .04 | .01 | n.a. | n.a. | n.a. | n.a. | -.09 | -.02 | .17^*^ | .12 | -.08 | .10 | .20 | .10 |
| **Expectancies regarding medication** |  |  |  |  |  |  |  |  |  |  |  |  |  |  |  |  |
| Expected effectiveness of medication [in general] for pain relief, r | .26^*^ | **.31^*^** | -.14^*^ | -.03 | n.a. | n.a. | n.a. | n.a. | **.30^*^** | .19 | -.23^*^ | -.05 | .07 | **.30^*^** | .10 | -.11 |
| Expected effectiveness of medication [in general] for itch relief, r | .24^*^ | .26^*^ | -.15^*^ | -.03 | n.a. | n.a. | n.a. | n.a. | .08 | -.03 | -.07 | -.07 | **.35^*^** | **.45^*^** | -.03 | -.02 |
| Expected side effects of medication [in general], r | -.23^*^ | -.21^*^ | .22^*^ | .07 | n.a. | n.a. | n.a. | n.a. | -.24^*^ | -.21 | .27^*^ | .13 | **-.42^*^** | **-.33^*^** | .24 | .22 |

*Note*: Gs1: General sample 1, Gs2: General sample 2, RAs: Rheumatoid Arthritis sample, ADs: Atopic dermatitis sample. GAMQ theoretical range of scores: Total 12 - 60 (12 items) Trust 4 - 20 (4 items), Concerns 3 - 15 (3 items), Reluctance 5 - 25 (5 items). n.a. = data not available; For ANOVAs:^*^p<.05, ^**^ p<.01, ^***^p<.001; For correlations: ^*^ bootstrapped confidence interval did not include 0. ^1^ In treatment for long-lasting medical or psychological complaints or diseases other than Ra for RAs and AD for ADs. SF12 PCS: short form 12 physical component score, SF-12 MCS: short form 12 mental component score, DAS44: disease activity score, POEM: patient oriented eczema measure. Medium and large effect sizes are printed in bold.
